# Supplementary material for: Discovery and pharmacophoric characterization of chemokine network inhibitors using phage-display, saturation mutagenesis and computational modelling
Source: Nat Commun. 2023 Sep 16;14:5763. doi: 10.1038/s41467-023-41488-z (PMC10505172; doi:10.1038/s41467-023-41488-z)
Supplement: Supplementary file 4 — Source Data [file 41467_2023_41488_MOESM4_ESM.zip › SourceData/scripts/xoctet_20230808081637.tar!/xoctet/analysis/kinetic/SV_E23_sHD2_CXCL13_kinetic_plot.pdf]

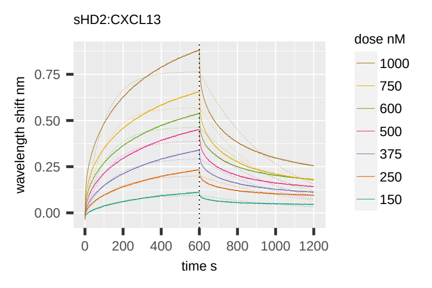

sHD2:CXCL13
dose nM
1000
750
600
500
375
250
150
wavelength shift nm
0.00
0.25
0.50
0.75
0
200
400
600
800
1000
1200
time s
